# Magnetic and Magneto-Optical Properties of Fe_75−x_Mn_25_Ga_x_ Heusler-like Compounds

**DOI:** 10.3390/ma13030703

**Published:** 2020-02-04

**Authors:** Daniel Král, Lukáš Beran, Martin Zelený, Jan Zemen, Roman Antoš, Jaroslav Hamrle, Jakub Zázvorka, Michal Rameš, Kristýna Onderková, Oleg Heczko, Martin Veis

**Affiliations:** 1 Faculty of Mathematics and Physics, Charles University, Ke Karlovu 5, 12 16 Prague 2, Czech Republic; daniel.kral@karlov.mff.cuni.cz (D.K.); beranlu@gmail.com (L.B.); zeleny@ipm.cz (M.Z.); antos@karlov.mff.cuni.cz (R.A.); hamrle@karlov.mff.cuni.cz (J.H.); zazvorka.jakub@gmail.com (J.Z.); 2 Faculty of Mechanical Engineering, Institute of Materials Science and Engineering, Brno University of Technology, Technická 2896/2, 616 69 Brno, Czech Republic; 3 Faculty of Electrical Engineering, Czech Technical University in Prague, Technická 2, 166 27 Prague, Czech Republic; zemenja1@fel.cvut.cz; 4 Institute of Physics of the Czech Academy of Sciences, Na Slovance 1999/2, 18221 Prague 8, Czech Republic; ramesm@fzu.cz (M.R.); navitas.onderkova@gmail.com (K.O.)

**Keywords:** Heusler compounds, Fe-Mn-Ga, martensitic transformation, Curie point, magneto-optics, ab initio

## Abstract

Fe_75−x_Mn_25_Ga_x_ Heusler-like compounds were investigated in a wide range of Fe/Ga ratios while keeping the Mn content constant and equal 25 at% in order to elucidate the interplay between magnetic properties and composition. Materials were prepared by arc-melting from pure elements and subsequently annealed. Experimental investigations were focused on magnetization behavior in a wide temperature range from 4 to 1000 K and magnetic field up to 9 T. Optical and magneto-optical (MO) measurements were employed to shed more light on the magnetic state and electronic structure of investigated materials. Magnetization measurements indicated that in the vicinity of stoichiometry (Fe_2_MnGa) the compounds are ferro/ferrimagnetic, whereas the Fe-deficient compound is paramagnetic and at high Fe concentration the antiferromagnetic interaction prevails. Theoretical calculations of corresponding ordered and disordered stoichiometric compounds were carried out and compared to the experiment on the level of net magnetic moment as well as magneto-optical spectra. This comparison suggests that the Heusler crystal structure, L2_1_, is not present even close to stoichiometry. Moreover, the comparison of density of states (DOS) for ordered and disordered structures allowed us to explain missing martensitic transformation (MT) in investigated materials.

## 1. Introduction

Some Heusler compounds undergo martensitic transformation (MT), which is essential for shape memory and caloric behavior [1]. Moreover, the twinned low symmetry phase can exhibit magnetically induced reorientation that manifests itself as a huge magnetic field-induced strain, more than twenty times larger compared to that of giant magnetostrictive materials [2,3,4]. Crucial for all effects is the presence of MT, which is due to a peculiar electronic structure in the vicinity of the Fermi level [5]. The main examples of magnetic shape memory (MSM) material are Ni-Mn-Ga Heusler compounds. However, these compounds suffer from several shortcomings such as fragility [6] and relatively low martensitic and ferromagnetic transition temperatures [7,8,9].

Heusler Fe-Mn-Ga compounds have been proposed as good candidates for new MSM material [10] as they have a much higher Curie point than Ni-Mn-Ga. However, it was found very soon that martensitic transformation is not a universal feature, and it occurs only in some particular compositions [11,12,13,14]. Apart from thermally induced transformation [15], even the field-induced transformation was observed for Fe_2_MnGa by Zhu et al. [16].

It was found that ternary compounds close to Fe_2_MnGa composition crystallize in various cubic structures defined by a different atomic order [12]. The ordering is very sensitive to compositional variation and even a very small deviation from stoichiometry, often negligible or even undetectable by usual EDS, can produce either bcc-like (disordered B2, ordered L2_1_ Heusler structure) or fcc-like (L1_2_ structure) phases or their mixture [12,17]. Apparently, thermal history can also play a role in establishing various phases [18].

Unlike Ni-Mn-Ga, Fe-Mn-Ga compounds contain two kinds of atoms with different magnetic moment localization but with comparable magnitude. Their interaction can provide various magnetic arrangements from antiferromagnetic to ferri- or ferromagnetic states. The close coexistence of the phases in a single compound is indicated by the exchange bias [19]. Such coexistence, in connection with a complex interplay of bcc- and fcc-like phases, results in complicated magnetic behavior. Overall, the magnetic state is not well understood even for stoichiometric Fe_2_MnGa. Despite the mixed structural order of fcc- and bcc-like phases in near stoichiometric Fe_2_MnGa, Kudryavtsev et al. were able to determine its electronic and optical properties and compare it with ab initio calculations [13,20]. However, the published results are incomplete as the most studied compositions are close to the stoichiometric Fe_2_MnGa compound.

Here we present a comprehensive study encompassing previous experimental reports and theoretical predictions. We investigated experimentally Fe_75-x_Mn_25_Ga_x_ compounds derived from Heusler compounds in a wide range of compositions with varied Fe/Ga ratios while keeping the Mn content constant and equal 25 at%. The magnetic and MO measurements are complemented with ab initio calculations of stoichiometric Fe_2_MnGa with different lattice structures and magnetic ordering in an attempt to clarify the observed complex behavior and differences between our experimental results and previous calculations.

## 2. Materials and Methods 

Polycrystalline bulk samples were prepared by arc-melting from pure elements in Ar atmosphere using a Buhler furnace. After the homogenization heat treatment at 1073 K/24 h, the samples were cut and polished mechanically and electrolytically to obtain high-quality surfaces for scanning electron microscopy (SEM) and optical and magneto-optical measurements. SEM was used to reveal the microstructure, phase composition, and grain size. We used SEM Tescan Fera 3 equipped with electron dispersion spectroscopy (EDS) analyser and electron backscattered diffraction (EBSD) device. The crystal structure was evaluated from EBSD. Composition of the polycrystalline buttons were determined by EDS with error up to 1 at% particularly on Mn content. The errors in these particular compounds were estimated from our analyses of Ni-Mn-Ga Heusler compounds, using comparison with X-ray fluorescence analysis with standards.

The magnetic properties with field and temperature were measured in the interval from 10 to 1000 K and field up to 9 T using a PPMS vibrating sample magnetometer in PPMS by Quantum Design. The spontaneous saturation magnetization was determined by extrapolating the high-field magnetization to zero field.

A magneto-optical spectrometer based on the rotating analyzer [21] method was obtained to acquire complex magneto-optical Kerr effect (MOKE) in polar configuration with nearly normal light incidence in the photon energy range from 1.2 to 4.5 eV. Applied magnetic field was 1.2 T to bring the samples close to the magnetic saturation. All magneto-optical data were measured at room temperature.

The Vienna Ab initio Simulation Package (VASP, ver. 5.4.4) was used for structural relaxation and calculations of magnetic moments, densities of states (DOS) as well as optical properties of stoichiometric Fe_2_MnGa [22,23]. In our calculation, the electron-ion interaction was described by projector augmented-wave (PAW) potentials [24,25]. We used the gradient-corrected exchange–correlation functional proposed by Perdew et al. [26]. To include expected disorder, the Special Quasirandom Supercell (SQS) [27,28] with 32 atoms as a model of disordered system was generated using a separate software tool [29]. This method is based on optimizing pair correlation coefficients to represent a statistically random solid solution.

## 3. Results and Discussion

Prepared materials ranged from the Fe_25_Mn_25_Ga_50_ to Fe_65_Mn_25_Ga_10_ keeping Mn content at 25 at% as close as possible. The chemical composition determined for most compounds by EDS is listed in Table 1 together with saturation magnetization and Curie temperature. The EDS analysis indicated that the nominal composition does not differ significantly from the measured one. Listed samples were further analyzed by magneto-optical measurements.

### 3.1. Structural Properties

**Figure 1:**
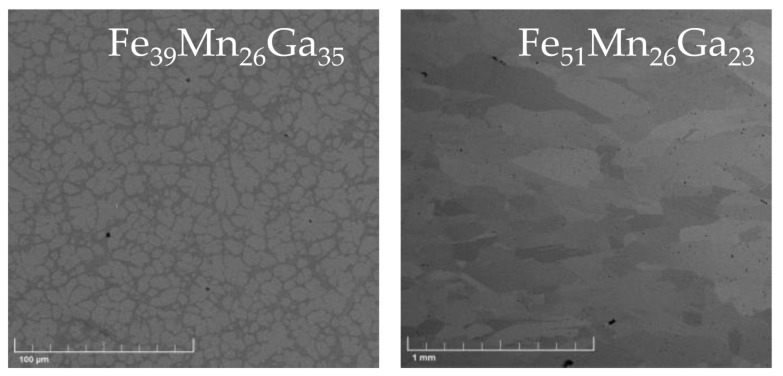
Microstructure of two-phase Fe40 (**left**) and single-phase Fe52 (**right**) samples observed by backscattered electrons in SEM. It clearly shows the two-phase character of the Fe40 sample showing irregular flower-like islands in a matrix. Sample Fe52 is single phase with relatively large grains. Composition determined by EDS is indicated in the figure.

All studied materials had a cubic structure at room temperature. None of the studied materials exhibited transformation to the lower symmetry phase, that is, martensitic transformation (as determined from magnetic measurement shown below) upon cooling. SEM observation using backscattered electrons revealed that two compounds, Fe65 and Fe52, were single cubic phase. The example of single-phase microstructure is shown in Figure 1 (right). SEM indicated a non-homogeneity of Fe50; however, different phases could not be resolved. A notable exception was the Fe40 compound, in which two different phases could be clearly identified, as shown in Figure 1 (left). The EBSD measurement indicated two cubic phases. These phases had very similar compositions as determined by EDS. Magneto-optical Kerr Effect (MOKE) microscopy indicated that only one phase was ferromagnetic at room temperature as the magnetic domains could be observed. The phase formed ferromagnetic islands immersed in a non-magnetic matrix. The two-phase character of the compound was confirmed by magnetization measurement (see below).

### 3.2. Magnetic Properties

**Figure 2:**
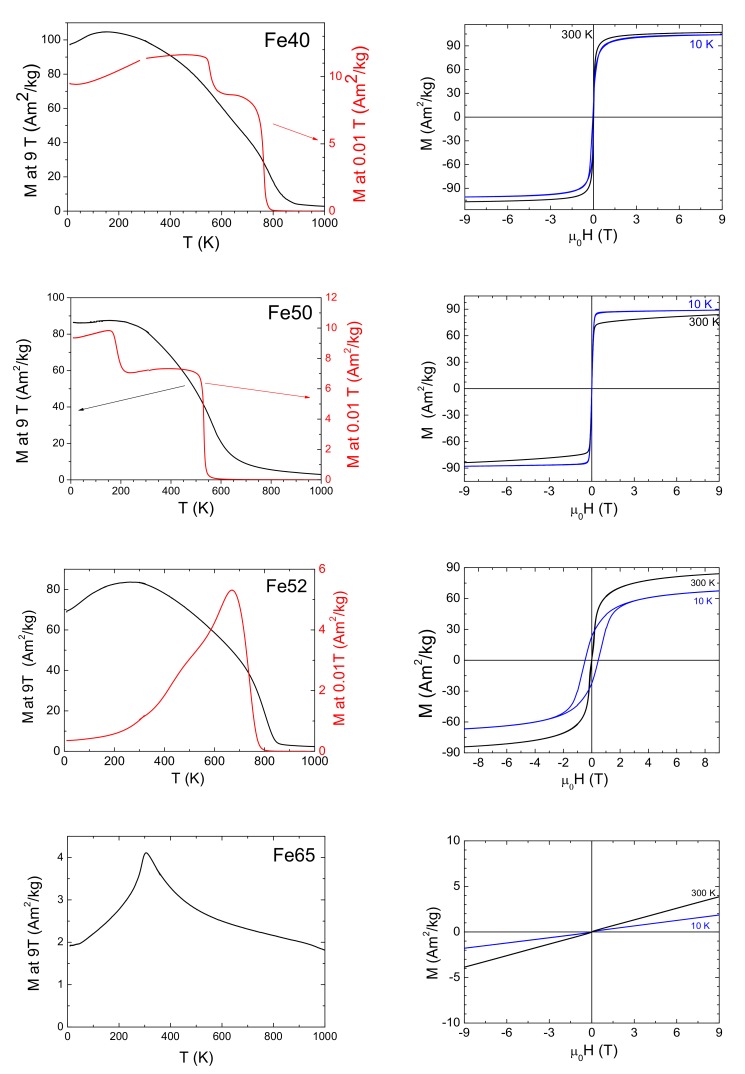
Magnetization curves at room and 10 K temperatures (**right**) and thermomagnetic curves measured upon heating (**left**) of selected samples marked in figure. Black line and left *y*-scale indicate saturation magnetization at 9 T. Red line and right *y*-scale indicate low-field magnetization (0.01 T).

Figure 2 summarizes the magnetization loops and temperature dependence of low-field and high-field magnetization of all magnetically ordered samples. The low-field magnetization confirmed expected high Curie temperatures in all compounds close to stoichiometry. The high Curie temperature phase can be identified from the literature as fcc phase [12,13]. The highest temperature, about 760 K, was measured for the sample (Fe50) closest to stoichiometry. The temperatures are listed in Table 1. The low-field magnetization measurement also confirmed the single phase in Fe52 and the two-phase character of Fe40 in agreement with SEM (Figure 1), indicating low Curie temperature (183 K) of the second phase. This agreed with MOKE observation at room temperature. According to Kudryavtsev, the matrix phase with a low Curie point is the disordered bcc phase [12,13].

Moreover, the low-field measurement revealed two different high Curie temperatures in Fe50 (as shown in the second panel of Figure 2), indicating, in accordance with inhomogeneities observed by SEM, that the sample contained two different phases. According to Kudryavtsev [12], it should contain fcc and bcc phases. Although the fcc phase exhibited a high Curie point in accordance with the literature, the expected bcc phase had quite a high Curie temperature compared to that of Fe40. It seems to exclude the presence of the bcc phase, but it may be also ascribed to different composition close to stoichiometry. On the other hand, the Curie temperature of the assumed fcc-phase is comparable with sample Fe52 in line with the literature [13]. High-field thermomagnetic curves support the described behavior; however, the separation between phases cannot be determined in any case.

Magnetization loops of selected samples at room temperature and close to 0 K, shown in Figure 2, indicate that the materials close to stoichiometry were all ferromagnetic or ferrimagnetic in agreement with [12]. With decreasing temperature, the saturation magnetization was often reduced below room temperature magnetization. The decrease of saturation magnetization with decreasing temperature is well illustrated by high-field thermomagnetic curves. This decrease indicated the onset of antiferromagnetic interaction below room temperature.

Interestingly, the Fe52 sample, which from SEM observation and low-field magnetic measurement seems to be single-phase material, exhibited a sharply increasing coercivity and a strong decrease of low-field magnetization with decreasing temperature. This behavior is not observed for any other compositions. The lowering susceptibility and increased coercivity can indicate enhanced magnetic domain pinning. It may be ascribed to antiferromagnetism in disturbed regions [12] as grain boundaries and even within antiphase boundaries [30,31,32] on which the magnetic domain walls can be pinned.

The compounds with the composition strongly deviated from stoichiometry were either paramagnetic or antiferromagnetic. Clear antiferromagnetic behavior was observed for Fe65 with Néel temperature T_N_ about 300 K, determined from the temperature dependence of saturation magnetization. This is similar to temperature, where the decrease of saturation magnetization is observed for other compositions. It suggests that the antiferromagnetic interaction becomes gradually stronger with increasing Fe content (i.e., from Fe40 to Fe65 samples).

**Figure 3:**
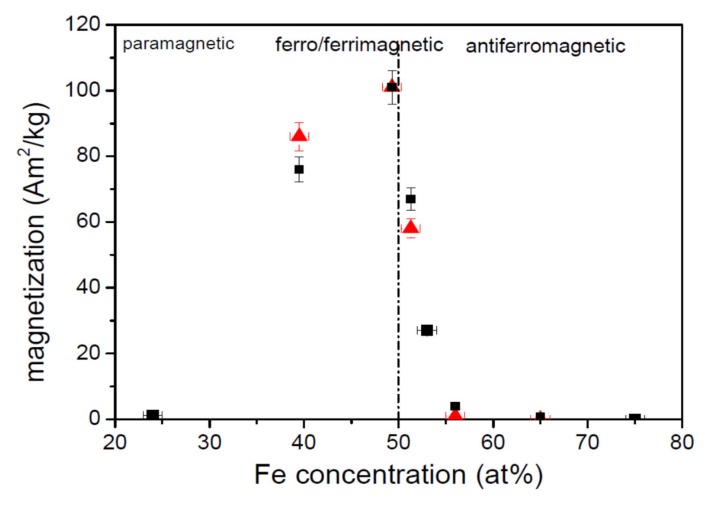
Spontaneous magnetization at 10 K obtained by extrapolation from high-field magnetization (up to 9 T) to zero field is represented by red triangles. Stoichiometric composition of Fe_2_MnGa is marked by the vertical broken line. Room temperature magnetization at 1.4 T related to MO measurements is represented by black squares. The estimated errors in composition and magnetization determination are marked.

The dependence of the spontaneous magnetization at 10 K on Fe content shown in Figure 3 summarizes previous observations. For low Fe concentration the material is paramagnetic, for higher Fe concentration, the ferromagnetic interaction is established and saturation magnetization increases. The maximum moment occurs at stoichiometry. With increasing Fe content above stoichiometry, the magnetization sharply dropped to almost zero due to antiferromagnetic interaction. The residual magnetic moment for the compound with high content of Fe can be due to not fully compensated antiferromagnetism or due to structural inhomogeneity, that is, the presence of a small volume of ferromagnetic phase or even a minute amount of pure Fe.

Room temperature magnetization measured at 1.4 T follows the trends observed for the spontaneous magnetization. It is also shown in Figure 3 as it is important for MO measurement done at room temperature.

### 3.3. Magneto-Optical Spectroscopy

**Figure 4:**
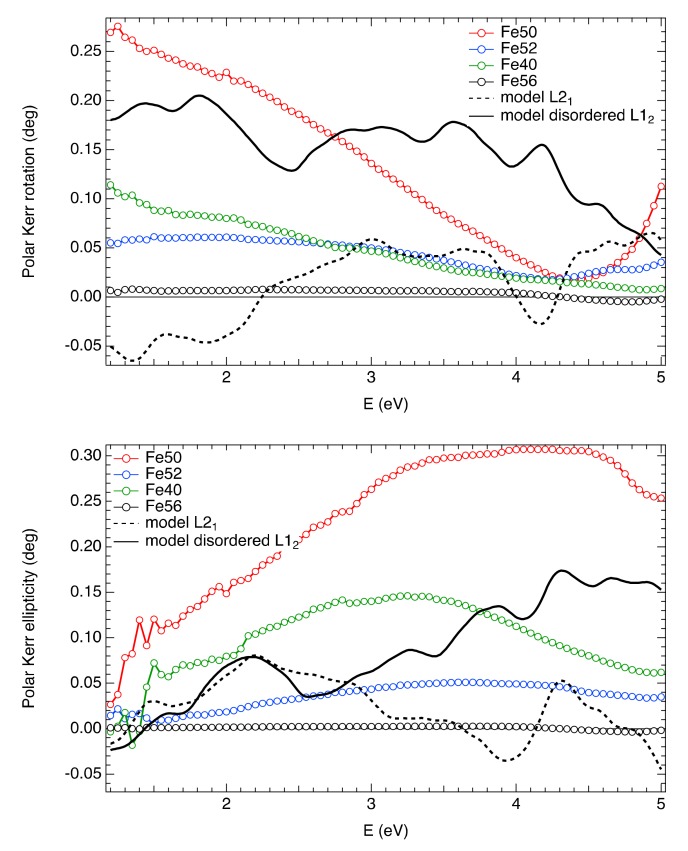
Experimental spectra of polar Kerr rotation (**top**) and ellipticity (**bottom**) measured at 1.2 T and room temperature together with ab initio theoretical calculations.

Room temperature experimental spectra of polar Kerr rotation and ellipticity are shown in Figure 4. The spectra of polar Kerr rotation exhibit monotonous behavior with an increase towards the low energy region and an indication of a spectral structure near 2 eV. On the other hand, the ellipticity spectra rise with increasing energy with a broad maximum situated near 3.5 eV.

This spectral dependence resembles already reported results on polycrystalline Fe_48_Mn_24_Ga_28_ [33] and Fe_50_Mn_25_Sn_25_ [34] bulk samples. However, the spectra were completely different than those previously reported on other Heusler compounds containing Mn, but without Fe atoms, such as Ni-Mn-Ga [35,36], Ni-Mn-Sn, Ni-Mn-Sb, or other compounds containing Mn atoms [37]. This result indicates a major difference in the electronic band structure between Fe-Mn-Ga and Ni-Mn-Ga compounds.

**Figure 5:**
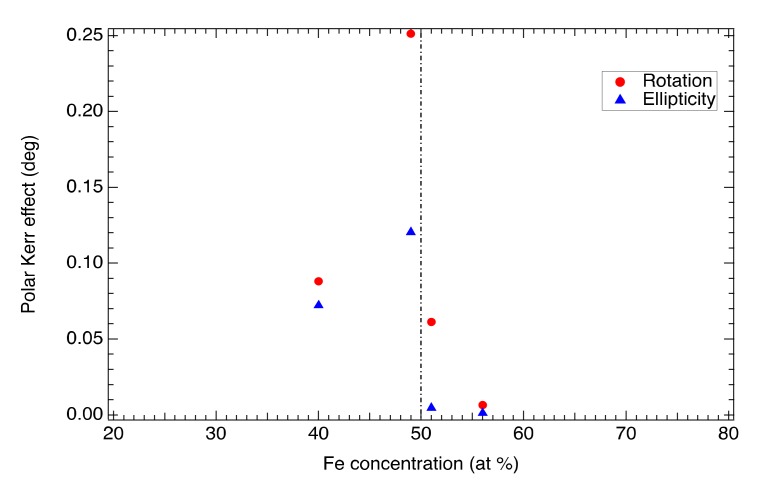
Composition-dependent polar Kerr effect of Fe-Mn-Ga compounds at the photon energy of 1.5 eV.

Figure 4 also shows strong variation of magneto-optical effect upon the composition of the sample. Although the spectral behavior is similar for all the samples, sample Fe50 exhibits the highest MOKE amplitude, whereas sample Fe56 has almost negligible magneto-optical response. This is consistent with magnetic measurements and is demonstrated in Figure 5, where the amplitude of MOKE at 1.5 eV is plotted as a function of Fe composition. As one can see from this figure, the magnetization together with MOKE has a maximum for nearly stoichiometric composition.

### 3.4. Theoretical Calculations

To explain our experimental data and to compare with previously published results, we performed new ab initio calculations with stoichiometric composition. Kudryavtsev et al. [13] show that the L2_1_ ordered structure has slightly higher total energy and thus it does not appear in the stoichiometric compound. This calculation seems to suggest that there is no Heusler compound with proper ordering. We next considered the L1_2_ (fcc) ordered structure.

Previous ab initio calculations performed for the stoichiometric compound with the L1_2_ (fcc) order indicated the ferromagnetic ordering as the most stable with a magnetic moment of 6.13 μ_B_/f.u. This is significantly higher than measured values of saturation magnetization of 4.23 μ_B_/f.u. In contrast, the ferrimagnetic ordering with opposite orientation of magnetic moments at Fe and Mn atoms exhibits much smaller magnetic moments of 0.48 μ_B_/f.u. [13].

In our calculation, we used the L1_2_ (fcc) structure described previously [13] but we considered opposite orientation of magnetic moment at neighboring Fe atoms within the same plane. This resulted in a magnetic moment of 1.90 μ_B_/f.u. An even higher magnetic moment of 2.75 μ_B_/f.u. was found for antiferromagnetic interaction between Fe planes, whereas in-plane interaction between Fe atoms was ferromagnetic. The magnetic moment is, however, still lower than experimentally observed values for nearly stoichiometric compounds.

Further increasing of magnetic moments can be achieved by considering the chemical disorder between Ga and Fe atoms in the L1_2_ lattice. We used the Special Quasirandom Supercell (SQS) [27,28] with 32 atoms as a model of disordered system. Such a supercell exhibits a magnetic moment of 4.31 μ_B_/f.u. due to the opposite orientation of magnetic moments at particular Fe atoms. Antiferromagnetic interaction appears if the Fe atom is surrounded at least by six other Fe atoms. In an ordered L1_2_ structure, each Fe atom is surrounded by only four other Fe atoms. Moreover, we also found that this ferrimagnetic state of disordered structure was energetically more favorable than ferromagnetic ordering about 0.017 eV/atom. This clearly indicates that the compound Fe50 is disordered L1_2_, which results in the highest magnetic moment of all ferrimagnetic states.

For slightly non-stoichiometric compounds with excess Fe content (i.e., the Fe52 sample), a sharp decrease of magnetic moment down to 2.4 μ_B_/f.u. was observed in the experiment. Based on the theoretical calculation of stoichiometric compound, this sharp decrease of magnetic moment can be explained by the disappearance of the disorder and the appearance of L1_2_ ordering. The theoretical prediction for ordered compounds is 2.75 μ_B_/f.u or 1.9 μ_B_/f.u., depending on the mode of antiferromagnetic interaction as described above. The further sharp decline of magnetic moment observed in the experiment in the compounds with higher Fe content can be ascribed to fully established antiferromagnetic interaction.

The ab initio calculation showed that further decrease of magnetic moment is due to increasing chance to form Fe clusters with oppositely oriented magnetic moments at Fe atoms. The predicted trend was observed in the experiment; however, the experimentally observed magnetization decrease is much sharper than predicted.

To explain the missing MT in the investigated samples, the ab initio calculations of MOKE spectra of L2_1_ and disordered L1_2_ structures were performed using the complex permittivity tensor obtained within the linear response theory as implemented in the VASP 5.4 code. The results are shown in Figure 4 together with experimental data. Theoretical spectra of L2_1_ structure do not follow experimental data, as they exhibit several maxima and minima and change sign of MOKE several times across the investigated energy range. This indicates the absence of L2_1_ order in the investigated samples.

On the other hand, the spectra of the disordered L1_2_ structure provide better agreement with the experiment. They exhibit similar monotonous increase and decrease of polar Kerr rotation and ellipticity, respectively. Admittedly, the amplitude of the effect is lower than the experimental data of the Fe50 sample. However, this can be explained by the relatively small unit cell (32 atoms) used in the calculation due to prohibitively high memory requirements. Such a small cell might describe the more severe deviation from the fully ordered structure than the disorder present in our sample. This deviation then changes the electronic structure excessively as manifested by the smooth DOS below, which can result in a suppression of the probability of electronic transitions responsible for magneto-optical response.

**Figure 6:**
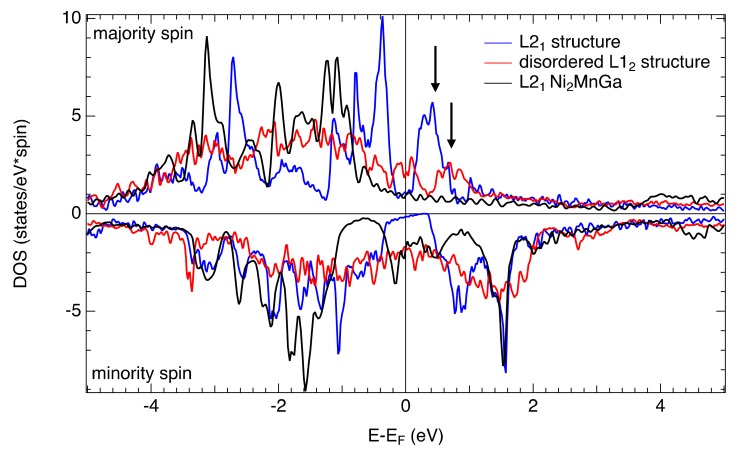
Calculated spin-resolved total DOS for L2_1_ and disordered L1_2_ structures compared to those of stoichiometric Ni_2_MnGa.

To explain the difference in the MOKE of stoichiometric Ni-Mn-Ga and investigated Fe-Mn-Ga samples, spin-resolved total DOS were calculated and are displayed in Figure 6.

Comparing the total DOS of L2_1_ and disordered L1_2_ structures with those reported for Ni_2_MnGa [5], one can see a major difference in the majority spin channel just above the Fermi level. While the L2_1_ and disordered L1_2_ structures of Fe_2_MnGa have a clearly visible band approximately 0.2–0.3 eV above the Fermi level (visible as a sharp peak indicated by arrows), this band is completely missing in the case of Ni_2_MnGa. It indicates that a substitution of Ni by Fe results in the presence of empty states above the Fermi level in the majority spin channel, which can act as excited states of particular electronic transitions. Figure 6 also shows that the disordered L1_2_ structure exhibits higher DOS at Fermi energy compared to the L2_1_ structure.

On the other hand, the minority spin channel looks similar for the case of Ni_2_MnGa [5] as well as for both Fe-Mn-Ga structures. Therefore, one should expect certain similarities in the MOKE spectra of these two compounds. Indeed, besides strong monotonous increase/decrease towards the low energy region, one can see certain indications of spectral structures in experimental MOKE spectra around 2 eV in rotation and 3 eV in ellipticity. These may originate, similarly to Ni_2_MnGa [35,36], from transitions between states in the minority spin channel. However, their transition probability is much lower compared to the low energy transitions. This is the case of the disordered L1_2_ structure where the energy gap in the minority spin channel around the Fermi level is not observed contrary to the L2_1_ structure and Ni_2_MnGa (see Figure 6). High DOS around the Fermi energy raise the optical absorption at lower energies [20] and enhance the magneto-optical response as was experimentally observed in the spectra of polar Kerr rotation. The huge difference in DOS in the vicinity of the Fermi level most likely suppresses the MT. Since the energy gap in the minority spin channel makes the martensite phase more energetically favorable over austenite [5] (it is shifted from below the Fermi level to the Fermi level during the MT), the stabilization of the disordered L1_2_ structure in the investigated compound is the reason for the missing MT.

## 4. Conclusions

In Fe-Mn-Ga compounds with constant 25 at% manganese content the saturation magnetization sharply grows, when iron content overcomes approximately 30 at% of Fe and then nearly immediately drops to zero above 50 at% of iron. The structural observation indicated that polycrystalline samples contained a mix of cubic phases, and the ordered single phase is difficult to prepare. We observed complex magnetic behavior ascribed to various magnetic ordering underlined by a different structural order. Our ab initio calculation showed that there is a transition between the disordered and ordered L1_2_ phases in the stoichiometric boundary (i.e., on Fe_2_MnGa). Inferring from this theoretical calculation, we suggest that the experimentally observed sharp drop of magnetization with increasing Fe content is caused by disorder–order transition in the L1_2_ structure and incipient antiferromagnetism in the ordered structure. The calculated magneto-optical spectra do not follow the spectral dependence reported for L2_1_ Ni_2_MnGa; however, they are consistent with the presence of the disordered L1_2_ structure. Disordered L1_2_ exhibits a completely different electronic structure near the Fermi energy and therefore does not undergo martensitic transformation.

## Figures and Tables

**Table 1:** Composition, spontaneous magnetization M_s_, and Curie temperatures T_c_ of selected compounds. The spontaneous magnetization in Bohr magnetons (μ**_B_**) per formula unit (f.u.) and the Curie temperature of the second phase are listed in brackets. The M_s_ was obtained by extrapolation the magnetization from high magnetic field to zero field. The error in determination of composition is about 1 at%; for magnetization measurement, the error is about 5%.

Name	Nominal	Determined by EDS	M_s_ at 10 K (Am^2^/kg) (μ_B_ per f.u.)	Tc (K)
Fe40	Fe40Mn25Ga35	Fe39.5Mn26Ga34.5	87 (3.77)	532 (183)
Fe50	Fe50Mn25Ga25	Fe49Mn26Ga25	100 (4.23)	762 (554)
Fe52	Fe52Mn25Ga23	Fe51Mn26Ga23	57 (2.40)	736 Single
Fe56	Fe56Mn25Ga19	Fe56Mn26Ga18	1 at 300 K (0.04)	Antiferromagnetic (traces of ferro.)
Fe65	Fe65Mn25Ga10	-	0.05 (0.02)	Antiferromagnetic T_N_ = 300 K
